# The intertropical convergence zone modulates intense hurricane strikes on the western North Atlantic margin

**DOI:** 10.1038/srep21728

**Published:** 2016-02-24

**Authors:** Peter J. van Hengstum, Jeffrey P. Donnelly, Patricia L. Fall, Michael R. Toomey, Nancy A. Albury, Brian Kakuk

**Affiliations:** 1 Texas A&M University at Galveston, Department of Marine Sciences, Galveston, Texas, 77554; 2 Texas A&M University, Department of Oceanography, College Station, Texas, 77843; 3 Woods Hole Oceanographic Institution, Department of Geology and Geophysics, Woods Hole, Massachusetts, 02543, USA; 4 University of North Carolina Charlotte, Department of Geography and Earth Sciences, Charlotte, North Carolina, 28223, USA; 5 U.S. Geological Survey, Eastern Geology and Paleoclimate Science Center, Reston, VA, 20192; 6 Antiquities, Monuments, and Museums Corporation/National Museum of The Bahamas, PO Box EE-15082, Nassau, The Bahamas; 7 Bahamas Underground, Marsh Harbour, Abaco Island, The Bahamas

## Abstract

Most Atlantic hurricanes form in the Main Development Region between 9°N to 20°N along the northern edge of the Intertropical Convergence Zone (ITCZ). Previous research has suggested that meridional shifts in the ITCZ position on geologic timescales can modulate hurricane activity, but continuous and long-term storm records are needed from multiple sites to assess this hypothesis. Here we present a 3000 year record of intense hurricane strikes in the northern Bahamas (Abaco Island) based on overwash deposits in a coastal sinkhole, which indicates that the ITCZ has likely helped modulate intense hurricane strikes on the western North Atlantic margin on millennial to centennial-scales. The new reconstruction closely matches a previous reconstruction from Puerto Rico, and documents a period of elevated intense hurricane activity on the western North Atlantic margin from 2500 to 1000 years ago when paleo precipitation proxies suggest that the ITCZ occupied a more northern position. Considering that anthropogenic warming is predicted to be focused in the northern hemisphere in the coming century, these results provide a prehistoric analog that an attendant northern ITCZ shift in the future may again return the western North Atlantic margin to an active hurricane interval.

Most intense North Atlantic hurricanes form just poleward of the Intertropical Convergence Zone (ITCZ) in the Main Development Region (MDR, 9°N and 20°N)^1^, where the dominant local environmental factor influencing hurricane activity is wind shear between the upper and lower troposphere^2^. The ITCZ is a zone of deep convection and heavy precipitation that is generated by convergence of the trade winds, and it migrates between 9°N and 2°N in response to seasonal sea surface temperature (SST) warming^3^. In the tropical Atlantic, the Atlantic Meridional Mode (AMM) is the dominant mode of ocean-atmospheric interaction between SSTs and low-level winds^4^. The AMM is associated with an anomalous meridional sea surface temperature gradient across the mean ITCZ latitude and a cross-gradient atmospheric boundary layer flow, which shifts the ITCZ towards the warmer hemisphere^4^,^5^,^6^. A positive phase of the AMM is associated with a strong meridional SST gradient, a northward shift in the ITCZ, and decreased vertical wind shear in the tropical Atlantic^7^. As such, meridional ITCZ displacements and intense hurricane landfalls on the western North Atlantic margin over millennial timescales could potentially co-vary during more positive AMM-like states of the tropical Atlantic. The Little Bahama Bank is an important geographic location for testing this hypothesis because it is located between sites on the western Atlantic margin in the Caribbean region^8^ and the US Eastern Seaboard^9^ where continuous hurricane reconstructions have been previously generated.

Blackwood Sinkhole is located 220 m inland from the shoreline on the eastern shore of Great Abaco in the northern Bahamas (Fig. 1; 26.79°N, 77.42°W). Sinkholes and blueholes are ubiquitous on the Bahamian landscape, and these develop from the subsurface dissolution and subsequent collapse of limestone bedrock^10^. The sediments within these karst basins are well-known archives of Holocene paleo climate records on carbonate landscapes^11^,^12^,^13^,^14^,^15^,^16^,^17^. Great Abaco is a low-lying carbonate island on the Little Bahama Bank, which has a typical tidal range of ~1 m. A wetland is currently adjacent to the sinkhole, and no streams discharge into the site (Figs 1 and 2). This groundwater-fed coastal basin is stratified, with anoxic saline groundwater located from 15 m below sea level to its maximum at 40 m below sea level (Figs 2 and S1). Since 1851 Common Era (CE), 42 hurricanes have passed within 65 nm of Blackwood Sinkhole. Sixteen of these storms were major hurricanes (Category 3, 4, or 5 on the Saffir-Simpson Scale), and the majority of these (88%) formed in the central and eastern MDR^18^.

## The Sediment Record

Two sediment push cores were collected from Blackwood Sinkhole using advanced technical scuba diving procedures. The sediment cores terminated on carbonate gravel, above which was laminated gyttja (~1 mm to 1 cm laminae) with discrete coarse-grained sedimentary layers (Fig. 3). Both laminations and coarse layers could be visually correlated between cores (e.g., BLWD-C1: 40 to 44 cm, BLWD-C2: 11 to 16 cm; Fig. 3), except for two non-laminated units of coarse terrestrial organic matter in BLWD-C1 (20 to 35 cm, and 84 to 93 cm, Fig. 3). Since these chaotic units likely represent slump events along the sinkhole periphery, the periodicity of coarse-grained sedimentation was examined further by developing a detailed age model from BLWD-C2 (See Supplementary Material). Episodic sedimentation occurred in BLWD-C2 from 7622 to 7494 Cal yrs BP_1950_ (2σ, 0.989 probability, 122 cm core depth) until 2960 to 2838 Cal yrs BP (2σ, 0.9089 probability, 109 cm core depth, Fig. S2). However, the sedimentation rate was nearly constant through the late Holocene (3000 years to present) from 109 to 0 cm (least squares regression: r^2^ = 0.991, *n* = 11 dates, 0.3 to 0.6 mm yr^−1^). Coarse-grained sedimentation was examined in contiguous 5 mm sediment samples downcore, which provides a record where each 5 mm sample represents 16 to 32 years over the late Holocene. Pollen was also analyzed in BLWD-C2 in 1 cm intervals downcore to further examine any relationship between coarse sedimentation into the sinkhole and vegetation changes on the adjacent terrestrial landscape.

The most obvious stratigraphic features are sand layers concentrated during discrete time intervals (Figs 2 and 3A): (a) 178 to 19 Cal yrs BP, (b) 438 to 387 Cal yrs BP, (c) 2486 to 1125 Cal yrs BP, and one event occurs at 2883 Cal yrs BP. Only mangrove invertebrates (e.g., gastropods, bivalves) and angular carbonate particles compose the sand layers. In contrast, rounded marine bioclasts and reef-dwelling foraminifera are abundant in the adjacent modern beach sediment (*n* = 5: mean 83% *Archaias*, Fig. S3). As such, the coarse sediment particles deposited at BLWD-C2 are most likely derived from the adjacent terrestrial and mangrove environments.

Local flooding combined with overwash from intense hurricane surge (≥category 3) most likely caused deposition of the coarse-grained horizons in Blackwood Sinkhole. Both numerical models^19^,^20^ and field observations^21^,^22^ indicate that since groundwater levels in low-lying unconfined aquifers are intimately linked to sea-level change and precipitation, landscape flooding often occurs during intense hurricane events. Indeed, the uppermost sand layer from 0.5 to 1.0 cm depth was deposited between 1981.7 and 2008.8 CE (2σ) when six hurricanes struck eastern Abaco, but only two generated local surge and flooding. Hurricane Floyd originated as an African easterly wave^23^ that made landfall on 14 September 1999 as a weakening category 4 event, with sustained 225 km/hr winds, +2 m storm surge and widespread flooding and destruction^24^. Hurricane Jeanne also originated as an African wave^25^, striking eastern Abaco Island on 25 September 2004 as a category 3 event with sustained winds up to 185 km hr^−1^. It is difficult to precisely attribute the event bed from 0.5 to 1.0 cm to a particular event because of the uncertainty in the age model, but the age of the deposit is consistent with deposition either from Floyd or Jeanne. Older coarse-grained layers deposited in the last 150 years may relate to other Bahamian hurricane disasters (e.g., 1932 CE hurricane, category 5), but uncertainty in the age model prevents confident association of other coarse-grained peaks to specific storm events. Based on the uppermost event, however, it appears that intense hurricane strikes with surge and flooding exceeding 2 m above sea level are responsible for event bed deposition (>20 mg cm^−3^) at this core locale.

The record does contain limitations. Multiple intense surge events occurring on sub-decadal timescales cannot be differentiated because of the sampling strategy (analytical time averaging) and sedimentation rate, and multiple types of storms can likely generate +2 m surges (e.g.,^26^). Also, the magnitude of coarse fraction sedimentation from one core alone cannot be used to infer hurricane intensity as multiple parameters (e.g., radius of maximum winds, storm translational velocity, local sediment budget and coastal geometry) can influence the lateral variability of a tempestite in the subsurface^27^. At the core site of BLWD-C2, however, varying coarse-grained sedimentation into Blackwood Sinkhole can be used to indicate active versus quiescent intervals of hurricane activity through time. Furthermore, the current geometry of the site was likely similar over the time that this sediment archive was accumulating, given the modest rates of regional sea-level rise during the late Holocene^28^ and the sinkhole’s structural geology.

## Discussion

### Climate forcing

The most significant result of the Blackwood reconstruction is the contrast between pronounced active versus quiescent intervals of intense hurricane landfalls based on event bed deposition, which closely matches previous evidence for intense hurricane strikes at Laguna Playa Grande in Puerto Rico (LPG, 18.09°N, 65.49°W, compare Fig. 4A,B)^8^. Relying on historical intense hurricane climatology for the last 164 years^18^, both LPG and Blackwood are predominantly vulnerable to storms forming in the central MDR. The concordance between the signal at LPG and Blackwood suggests that we sampled a similar population of intense hurricanes striking the western North Atlantic margin over the last 3000 years, which most likely originated in the central MDR.

Woodruff *et al.*^29^ demonstrated that the active interval from 2500 to 1000 Cal yrs BP was at least partially driven by changes in regional hurricane climatology, despite lower sedimentation rates in the older part of the LPG reconstruction. Furthermore, quiescent intervals observed at LPG were statistically unlikely under current climatological conditions, and could not be replicated even when climate conditions for a hurricane downscaling model were forced into a constant El Niño state^29^, which is thought to limit Atlantic hurricane activity^30^. Given the constant sedimentation rate in the record from Blackwood Sinkhole, and its replication of the LPG reconstruction, the pronounced oscillation between active versus quiescent periods of intense hurricane activity is most likely driven by broadscale climate features operating on multi-decadal or greater timescales, not interannual forcing like El Niño/Southern Oscillation.

In the modern climate, positive (negative) Atlantic Meridional Mode (AMM) phases are associated with a northward (southward) shift of the ITCZ, which reduces (increases) vertical shear in the MDR by moving the ascending branch of the Hadley circulation slightly northwards (southwards)^6^,^7^,^31^. In turn, a more northerly (southerly) position of the ITCZ enhances (diminishes) cyclogenesis in the eastern tropical North Atlantic (northeastern seaboard)^7^. Given that the AMM is excited on multi-decadal timescales by the Atlantic Multidecadal Oscillation^32^, a multi-decadal hurricane reconstruction may provide insight into likely instances of more common positive AMM-like states of the tropical Atlantic, and an attendant northern ITCZ displacement. During the Holocene, the temperature contrast between the Northern and Southern Hemisphere extratropics correlates well to Holocene-scale records of global hydroclimate and meridional ITCZ displacements^3^,^33^, which can be used to probe the long-term correlation between ITCZ migrations and western North Atlantic margin hurricane landfalls.

No intense hurricane is recorded at Blackwood from 2900 to 2500 Cal yrs BP, which is likely the tail-end of the quiescent period previously observed at LPG from 3800 to 2500 Cal yrs BP. This quiescent period is coeval with proxy-based evidence for a more southerly position of the ITCZ. For example, the temperature contrast between the extratropics in the Northern and Southern hemispheres suggests a more southern ITCZ position (Fig. 4D), which correlates well with terrestrial proxies of discharge into the Cariaco Basin^34^ and the Indian Monsoon^3^. Elsewhere, the stable carbon isotopic values preserved in a speleothem from Cold Air Cave in Northeastern South Africa are thought to represent the regional proportion of C_3_ vegetation (woodland savannas) versus C_4_ vegetation (grasses, aridity-adapted) on the landscape related to regional aridity^35^. The negative carbon isotopic excursion from 2900 to 2500 Cal yrs BP in Cold Air Cave indicates C_3_ vegetation expansion, which suggests increased regional moisture delivery from a more southerly ITCZ in Africa (Fig. 4E). Similarly, Laguna Pallcacocha in Ecuador documents a increase in intense precipitation events at this time (Fig. 4F)^36^. Previously, the Laguna Pallcacocha record was interpreted as a proxy for only El Niño-driven flooding events^8^,^36^, but here we suggest that increases in magnitude and frequency of red-hued beds deposited in Laguna Pallcacocha are also significantly impacted by southern ITCZ displacements.

From 2500 to 1000 Cal yrs BP, the North Atlantic experienced an interval of intense hurricane activity. This interval is coeval with paleoclimate evidence for a more northern ITCZ position relative to present, but not as far north as during the middle Holocene (Fig. 4D). Both Blackwood and LPG document this active interval, and hurricane-mediated export of coarse-grained sediment off the Grand Bahama Bank also increased^37^. The tail-end of this active interval is observed at Salt Pond, Massachusetts (Fig. 4C), where increased intense hurricane landfalls occur between 1700 and 900 Cal yrs BP^9^. Concurrent with the onset of this active interval is a prominent increase in the temperature contrast between the Northern and Southern Hemisphere extratropics at 2500 Cal yrs BP, which suggests an abrupt northerly displacement of the ITCZ (Fig. 4D). This begins a period of Caribbean aridity relative to the middle Holocene as inferred from terrestrial vegetation in both Hispaniola^38^,^39^,^40^ and Andros^15^, C_4_ plants expanded in South Africa^35^, and decreased intense precipitation events at Laguna Pallcacocha (Fig. 4G). Given the rapid ~1.5 °C warming observed in the eastern equatorial Atlantic during this interval^41^, oceanographic conditions would likely have been conducive for cyclogenesis. These records collectively suggest an abrupt shift to a more northerly ITCZ position from 2500 to 1000 Cal yrs BP, which would have favored cyclogenesis in the central MDR by reducing vertical wind shear, and increasing the likelihood of intense hurricane strikes in the western North Atlantic.

At 1000 Cal yrs BP, intense hurricane activity decreased in the western North Atlantic margin as evidenced at Blackwood, LPG, and Salt Pond. Previously completed statistical models based on ocean-atmospheric forcing of hurricane activity also projected a decrease in Atlantic hurricane activity after 1000 Cal yrs BP^42^. As discussed elsewhere^43^, poor definition of the ITCZ position in the expansive Amazon basin partly explains observed differences between the Ti-runoff proxy into the Cariaco Basin and other records of South American precipitation anomalies during the last millennium. However, a southern ITCZ displacement at 1000 Cal yrs BP could have increased precipitation and resultant accumulation rates of the Quelccaya Ice Cap^43^; allowed C_3_ vegetation to expand in South Africa^35^, and increased the likelihood of extreme precipitation events at Laguna Pallcacocha in Ecuador^36^. Further north, a southern displacement of the ITCZ at 1000 Cal yrs BP likely increased aridity at multiple sites in the northern subtropics such as in the Yucatan^44^, Dominican Republic^45^, and Cuba^46^, which could have decreased cyclogenesis in the MDR.

Late Holocene terrestrial vegetation changes in Abaco, reconstructed from pollen that was also preserved in BLWD-C2, provides further evidence for regional precipitation changes that are likely associated with meridional ITCZ displacements. Modern Bahamian precipitation is generally characterized by a latitudinal precipitation gradient^47^, in which only the northern islands are sufficiently mesic to currently support *Pinus* forests (Abaco, Grand Bahamas, Andros, and New Providence). Bahamian islands further to the south are more arid, in part due to their close proximity to regional low-level atmospheric subsidence between the northeasterlies and anticyclonic flow^47^. Furthermore, seasonal intensification of the subtropical ridge during boreal summer decreases Bahamian rainfall^48^ by increasing subsidence and intensifying the easterlies, creating the mid-summer drought. These atmospheric processes are necessarily linked to displacements of the ITCZ given their spatial relationship with the Hadley Cell^49^.

A wholesale change in regional moisture balance must have occurred at ~1000 Cal yrs BP when Abaconian forests transitioned from arid-adapted palms (Arecaeae) and tropical hardwoods (primarily *Bursera, Metopium,* Myrtaceae), to centennial-scale oscillations between the more mesic *Pinus caribaea* and *Conocarpus erectus* (buttonwood) in the last millennium. *Pinus* (pine) forests only became established on the northern Bahamian landscape ~700 years ago, based on pollen reconstructions from Abaco Island (Fig. 4H) and on nearby Andros Island^15^. The abrupt vegetation changes beginning at 1000 Cal yrs BP are also coincident with a loss of Abaco’s reptile-dominated terrestrial food webs^50^, and perhaps other mammals^51^. The earliest human remains in the northern Bahamas date between 1050 and 920 Cal yrs BP^52^, but the concurrent expansion of *Pinus* in Andros^15^ and Abaco at ~700 Cal yrs BP suggests a synchronous regional response to hydroclimate change. Perhaps modest rates of late Holocene sea-level rise expanded suitable habitat for *C. erectus* in the wetlands adjacent to Blackwood sinkhole over the last 1500 years, but not the observed rapid centennial-scale oscillations in the dominance between coastal mangroves versus interior species (pines, hardwoods) that begins at 1000 Cal yrs BP. Prior to 1000 Cal yrs BP, a more northerly positioned ITCZ could have shifted the zone of regional subsidence between the easterlies and anticyclonic flow northwards, in turn promoting a more arid northern Bahamian region.

The southern displacement of the ITCZ at 1000 Cal yrs BP that decreased hurricane activity appears to have first promoted *C. erectus* expansion from 900 Cal yrs BP, but was soon followed by rapid *Pinus* expansion at 700 Cal yrs BP (Fig. 4H). A Buttonwood-dominated wetland also expanded in southeastern Abaco at ~900 Cal yrs BP^53^. It appears that *C. erectus* populations already on the Bahamian landscape first benefited from the changing hydroclimate at 1000 Cal yrs BP. As regional subsidence between the anticyclonic flow and the easterlies concomitantly shifted southward with the ITCZ at 1000 Cal yrs BP, the resultant increased moisture delivery could have expanded suitable habitat in the topographic lows to the east of the study site. Thereafter, a slightly more northerly ITCZ from 700 to 500 Cal yrs BP is documented by the extratropical interhemispheric temperature anomaly and decreased accumulation rate of the Quelccaya Ice Cap (Fig. 4F), but not so far north as to return the northern Bahamas to arid conditions experienced from 2500 to 1000 Cal yrs BP. The southern ITCZ shift at 1000 Cal yrs BP seems to have initiated the necessary mesic conditions for *Pinus* expansion in both Abaco and Andros^15^.

**Figure 1:**
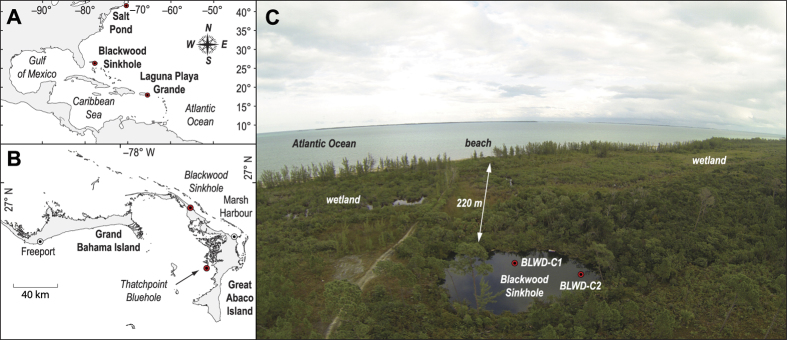
(**A**) The western North Atlantic region noting the location of Blackwood Sinkhole on the Little Bahama Bank, Laguna Playa Grande in Puerto Rico, and Salt Pond in Massachusetts, USA. (**B**) Islands on the Little Bahama Bank, and the position of Blackwood Sinkhole relative to Thatchpoint Bluehole. (**C**) Aerial photograph of Blackwood Sinkhole facing a northeasterly direction. Maps in (**A**,**B**) modified with permission after^13^.

**Figure 2:**
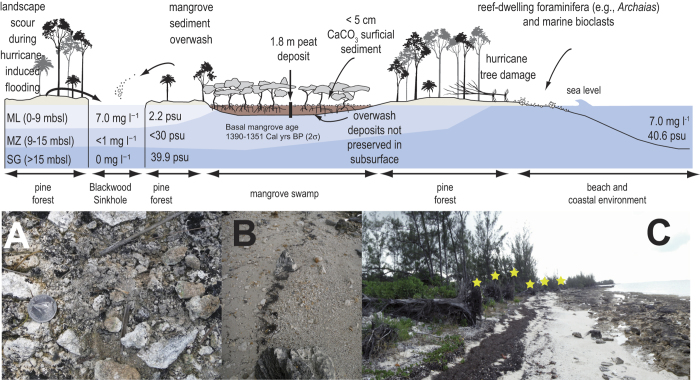
A conceptual model of the landscape surrounding Blackwood Sinkhole that illustrates the local sediment supply, and the likely processes that are promoting overwash into the sinkhole during an intense hurricane event (not to horizontal or vertical scale). Blue shading refers to different groundwater masses in the sinkhole, with their respective differences in dissolved oxygen and salinity (ML: meteoric lens, MZ: mixing zone, SG: saline groundwater). The peat in the adjacent mangrove swamp did not contain overwash deposits, and basal peat sediments were aged to ~1350 years ago (Table S1). Photographs: (**A**) The surficial sediment on the surrounding terrestrial landscape (the location of S1 in Fig. S4A) contains angular carbonate particles (weathered karst fragments) and no shell material. These terrestrial sediment particles are similar to the coarse-grained particles in the overwash deposits preserved in the sediment core from Blackwood Sinkhole (BLWD-C2). The diameter of the quarter is 24.2 mm. (**B**) In contrast, coarse sediment (>63 μm, 5 cm^3^) on the adjacent beach contains rounded marine mollusk fragments, foraminiferal assemblages dominated by reef-dwelling foraminifera (*Archaias angulatus*), and coral fragments. (**C**) A row of toppled trees (yellow stars, *Casuarina* sp.) from a previous storm, just behind the high-tide wrack line.

**Figure 3:**
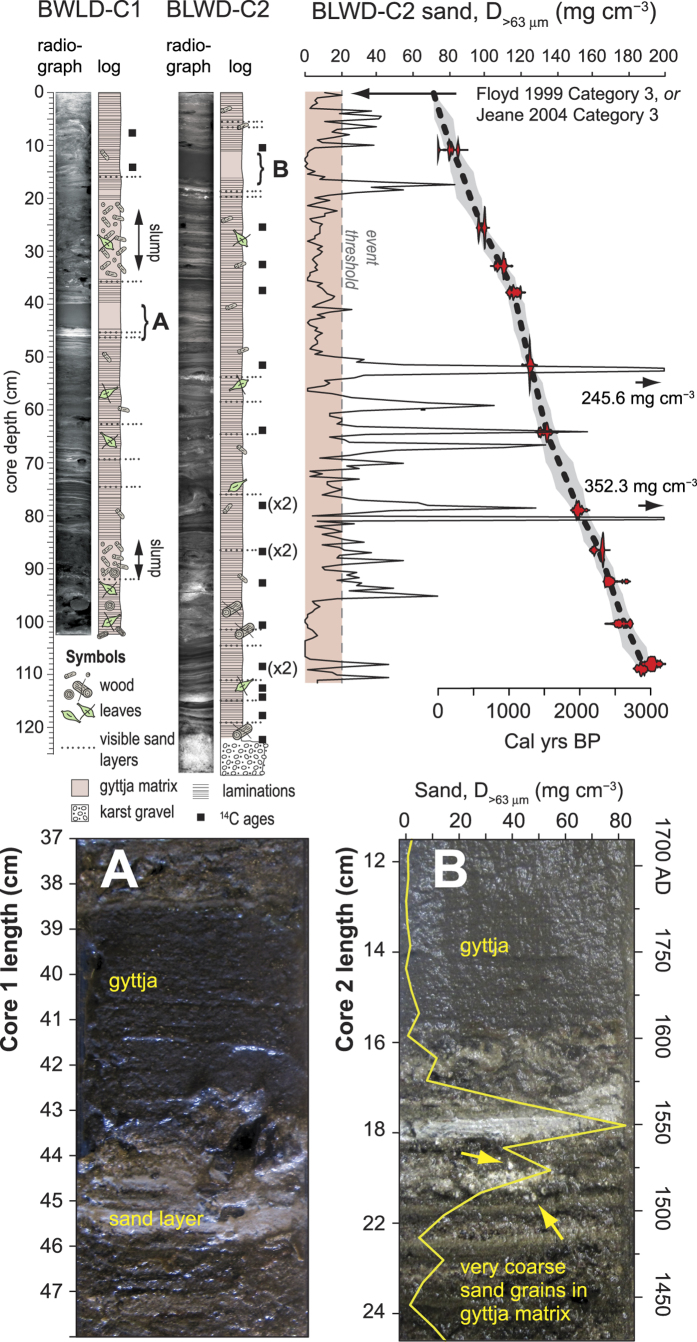
Core photographs, X-radiographs, generalized lithology, and position of radiocarbon dates (Table S1). The bottom panels (**A,B**) compare a salient algal gyttja horizon, laminations, and coarse layers between the cores. The top and bottom sand layers in panel (**B**) were deposited at 1549 AD (2σ: 1402 to 1715 AD) and 1524 AD (2σ: 1378.9 to 1698.8 AD), respectively.

**Figure 4:**
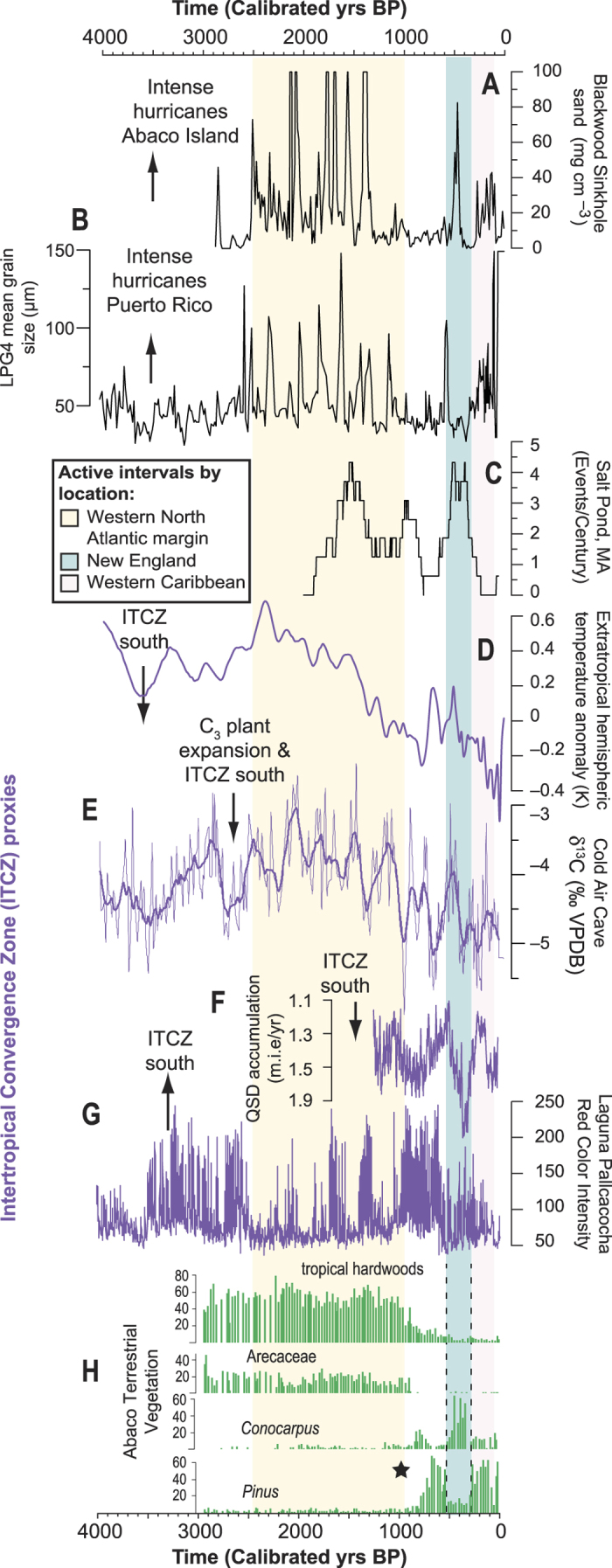
(**A**) Sand deposition into Blackwood Sinkhole from BLWD-C2, note that the vertical scale is truncated, (**B**) intense hurricane overwash deposits, Puerto Rico^8^, (**C**) Intense hurricane events at Salt Pond, Massachusetts^9^, (**D**) Northern-to-Southern hemisphere temperature anomaly^3^,^33^, (**E**) Raw (light purple) and 100-pt mean (dark purple) of δ^13^C measurements from speleothem T7 from Cold Air Cave, South Africa^35^, (**F**) Quelccaya Ice Cap accumulation rate, Peru^43^, (**G)** Laguna Pallcacocha red color intensity provides evidence for periods of increased intense precipitation, Ecuador^36^, (**H**) pollen-based evidence for vegetation changes on Great Abaco Island. Star denotes oldest radiocarbon dated human remains yet recovered from northern Bahamas^52^. Zero on the horizontal axis is 2000 CE.

From 600 to 300 Cal yrs BP, intense hurricane landfalls shifted to the North American northeast coast as recorded at Salt Pond, despite a more southerly ITCZ position. In the Bahamas, Blackwood records two intense events at ~500 Cal yrs BP, but a prominent active interval is recorded at Thatchpoint Bluehole (Fig. 1B)^13^. This is likely related to the lower intensity threshold for overwash deposition in the submerged bluehole (Thatchpoint) versus the subaerial sinkhole (Blackwood). The overwash record from Salt Pond in Massachusetts indicates one of the greatest active intervals in the American Northeast in the last 2000 years. On the Abaco landscape, there is a notable decline in *Pinus* synchronous with an increase in *C. erectus* at this time. This suggests expanded regional wetland development from increased moisture delivery, perhaps from the least exposure to the regional subsidence that currently promotes modern aridity on southern Bahamian islands^47^.

These local vegetation changes are consistent with evidence for a southernmost ITCZ displacement during the late Holocene based on the greatest accumulation rates of the Quelccaya Ice Cap in the last 1300 years^43^ and the wettest period observed at Laguna Pumacocha in South America^54^. A southerly ITCZ position should have hampered hurricane activity in the MDR. Indeed, Donnelly *et al.*^9^ attribute the increased hurricane strikes in the northeast between 600 and 300 Cal yrs BP to increased cyclogenesis or tropical transition of mid-latitude disturbances off the North American eastern seaboard in response to a western Atlantic warming event, which is likely related to a short-lived increase in overturning circulation^55^. Given its geographic position, it appears that the northern Bahamas were also impacted by the increased storm activity on the northeastern American seaboard during this interval. Blackwood Sinkhole only records two intense events, but Thatchpoint Bluehole documents a more prolonged active interval from likely weaker storms just developing.

From 300 to 100 Cal yrs BP a noteworthy increase in intense hurricane landfalls occurred at Blackwood Sinkhole that is coincident with decreased hurricane activity in New England. This active interval was observed previously at LPG, but higher sedimentation rates at LPG during the last several hundred years perhaps enhanced a bias for over-counting intense events in the last ~400 Cal yrs BP versus the earlier part of the record^8^. Due to its steady sedimentation rates, the reconstruction from BLWD-C2 suggests that this is a real feature of late Holocene hurricane activity. These results are at odds with other hurricane reconstructions that indicate that the Gulf of Mexico^11^ and Yucatan coast^12^ are inactive at this time. This interval occurs when the ITCZ should be positioned in a more southerly position during the Little Ice Age. A high-resolution coral-based SST reconstruction from the northern Bahamas suggests increased SSTs in at least part of the Atlantic Warm Pool at this time^56^, which may be impacting regional hurricane activity. These regional differences highlight the need for additional sub-decadal paleo hurricane records over the last millennium to more clearly resolve the regional timing and ocean-atmospheric forcing of paleo hurricane activity.

### Implications

This study indicates that centennial-scale shifts in the ITCZ have likely played an important role in modulating intense hurricane landfalls along the western North Atlantic margin over the last 3000 years. However, the results further emphasize a pattern of zonal variability in paleo hurricane landfalls on millennial timescales^9^, as evidenced by active intervals in the Gulf of Mexico^11^ versus western Atlantic margin (this study), which requires further evaluation. Considerable uncertainty still surrounds how North Atlantic hurricane activity will respond to anthropogenic influences on global climate^57^, with some modeling results predicting an increase in activity in the Atlantic basin^58^,^59^. Indeed, modern observations indicate that many variables influence hurricane intensity and frequency. As the Earth warms over this century, however, a northward shift in the thermal equator is predicted that will have an attendant ITCZ displacement^60^,^61^. The results presented here perhaps foreshadow a return to more persistent hurricane activity similar to a positive AMM-like state. The active interval of intense hurricane activity from 2500 to 1000 Cal yrs BP may provide an important analog for evaluating future hurricane and flooding risk along the now heavily-populated western North Atlantic margin.

## Methods

Aerial photograph of Blackwood Sinkhole (Fig. 1) was collected with a DJI Phantom 2 equipped with a GoPro Hero^3^ camera. Volumetric aliquots of sediment (2.5 cm^3^) at contiguous 5 mm intervals downcore were first rinsed over nested 32 μm and 63 μm meshes to isolate coarse silt- and sand-sized sediment fractions, respectively, and then desiccated overnight at 55 °C. Each sieved sediment fraction was then heated to 550 °C for 4.5 hours to combust organic particles. Finally, the remaining residue was re-weighed, and the volumetric quantity of coarse, inorganic sediment particles was obtained by difference (Sieve-first LOI method). The final measurement is mass (mg) of coarse particles >63 μm per cm^3^ of sediment [i.e.: D_> 63 um_ (mg cm^−3^), not density]. The chronology was developed with 21 accelerator mass spectrometer ^14^C dates (see Table S1) on terrestrial plant macrofossils. Conventional ^14^C ages were calibrated to calendar years with INTCal13^62^. Near linear sedimentation described the topmost 110 cm of BLWD-C2 (least squares regression: r^2^ = 0.991, *n* = 11 dates, Fig. S2), but *Bacon (v2.2)* generated the final age model from 110 to 0 cm using the IntCal13 calibration curve^63^.

Sediment sub-samples (*n* = 114, 1.25 cm^3^) were processed for pollen analyses using standard palynological techniques at approximately 1 cm intervals throughout BLWD-C2. Two *Lycopodium* spore tablets (University of Lund, Batch No. 124961, ~12,500 spores each) were added to each sample to allow calculation of pollen and spore concentration. Samples were screened through 180 μm mesh screens to remove larger organics (i.e., leaves and wood). The samples were processed following standard techniques with 10% HCl, 48% HF, 10% KOH, followed by acetolysis mixture^64^. The samples were then mounted on microscope slides, pollen grains were counted at 400–1000X magnification, and individual grains were identified with published taxonomic keys^64^,^65^,^66^,^67^ and the reference collection of P.L.F. An average of 360 pollen grains were counted in each sample. However, in the upper 31 cm where *Pinus* pollen dominates, at least 200 non-pine grains were counted and the sample mean equaled 492 grains (range: 433–673). From 32–109 cm, the mean was 320 grains (250–592). A final terrestrial pollen sum was calculated by excluding ferns, Cyperaceae, fungal spores and aquatic taxa due to the highly variable numbers and possible over representation.

## Additional Information

**How to cite this article**: van Hengstum, P. J. *et al.* The intertropical convergence zone modulates intense hurricane strikes on the western North Atlantic margin. *Sci. Rep.* **6**, 21728; doi: 10.1038/srep21728 (2016).

## Supplementary Material

Supplementary Information
