# Supplementary material for: The intertropical convergence zone modulates intense hurricane strikes on the western North Atlantic margin
Source: Sci Rep. 2016 Feb 24;6:21728. doi: 10.1038/srep21728 (PMC4764861; doi:10.1038/srep21728)
Supplement: Supplementary Information [file srep21728-s1.pdf]

The intertropical convergence zone modulates intense hurricane strikes on the western North Atlantic margin

Peter J. van Hengstum[1*], Jeffrey P. Donnelly[2], Patricia L. Fall[3], Michael R. Toomey[4], Nancy A. Albury[5], Brian Kakuk[6]

1. Texas A&M University at Galveston, Department of Marine Sciences, Galveston, Texas, 77554
2. Woods Hole Oceanographic Institution, Department of Geology and Geophysics, Woods Hole, Massachusetts, 02543, USA
3. University of North Carolina Charlotte, Department of Geography and Earth Sciences, Charlotte, North Carolina, 28223, USA
4. U.S. Geological Survey, Eastern Geology and Paleoclimate Science Center, Reston, VA, 20192.
5. Antiquities, Monuments, and Museums Corporation/National Museum of The Bahamas, PO Box EE-15082, Nassau, The Bahamas
6. Bahamas Underground, Marsh Harbour, Abaco Island, The Bahamas.
*Corresponding author: vanhenp@tamug.edu

Additional Sedimentary Information

Blackwood Sinkhole presents as a classic sinkhole basin with a stratified water column. The sinkhole provides a porthole into the local coastal aquifer, where the upper meteoric lens is stratified from the anoxic saline groundwater (Fig. S1). The anoxic conditions at the sediment water interface enhances sediment preservation by limiting the action of bioturbating organisms (e.g., worms, bivalves). Sedimentation rate at the site of BLWD-C2 has been nearly constant over the last 3000 years (Fig. S2, Table S1). Likely sediment sources include organic matter from primary production, authigenic calcium carbonate precipitation, and erosional products from the adjacent landscape. Despite the proximity of the sinkhole to the beach, classic beach sedimentary particles (rounded marine mollusks, reef-dwelling benthic foraminifera) were not found in the coarse layers of BLWD-C2 (Fig. S3).

One sediment push core was collected from the adjacent mangrove swamp, which sampled the entire peat stratigraphy to the eolianite bedrock (Fig. S4, BLWD-MC1, 26.799067°, −77.422352°). Before coring, we used a sediment probing staff to map the peat-eolianite contact in the subsurface to ensure we collected the most complete and expansive stratigraphic succession from this area. We also excavated several small pits in the mangrove peat. As such, the purpose of this additional sampling was to answer the following questions that are related to the overall sediment reconstruction from BLWD-C2: (1) are any overwash deposits preserved in the mangrove peat, and (2) how does the constant sedimentation rate at Blackwood Sinkhole over the last ~3000 years relate to timing of colonization of the mangrove swamp in the adjacent eolianite interdune swale?

The radiocarbon age of a terrestrial plant macrofossil at the base of the mangrove peat succession (BLWD-MC1: 108-110 cm) suggests that sedimentation in the interdune swale initiated at >1350 Cal yrs BP (Table S1). This is within ~200 years of the increase in *Conocarpus* pollen preserved in BLWD-C2, and likely expansion of the wetland environment adjacent to Blackwood Sinkhole. These results suggest that the near linear sedimentation at the coring site of BLWD-C2 has been maintained by several factors (i.e., not just organic sediment derived from mangroves) over the late Holocene. Indeed, additional cores are required from Blackwood Sinkhole to determine the complete impact of mangrove development on the spatial stratigraphic architecture in Blackwood Sinkhole over the late Holocene, which is beyond the scope of the present study.

Note: Any use of trade, firm, or product names is for descriptive purposes only and does not imply endorsement by the U.S. Government.

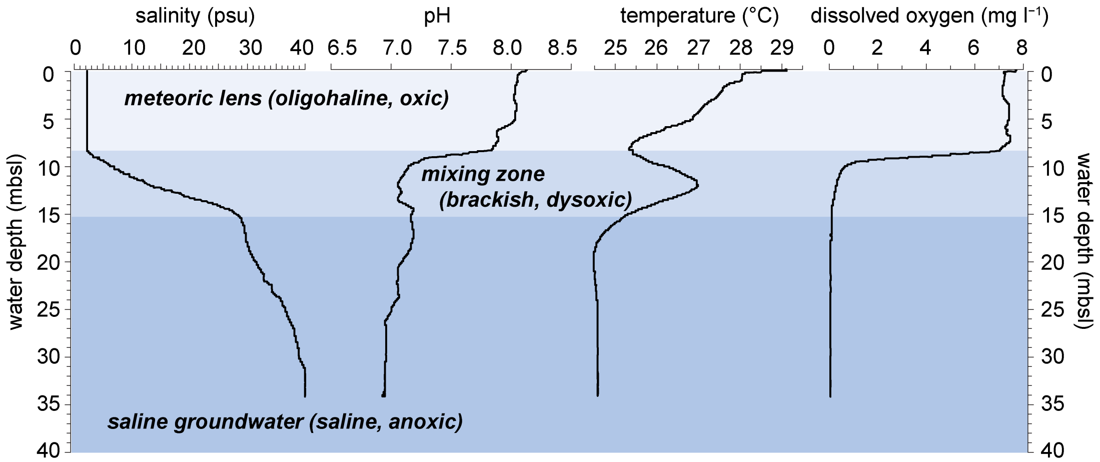

Figure S1. Vertical hydrographic profiles for Blackwood Sinkhole, measured on the 25 May 2014 with a YSI EXO1 multi-parameter sonde.

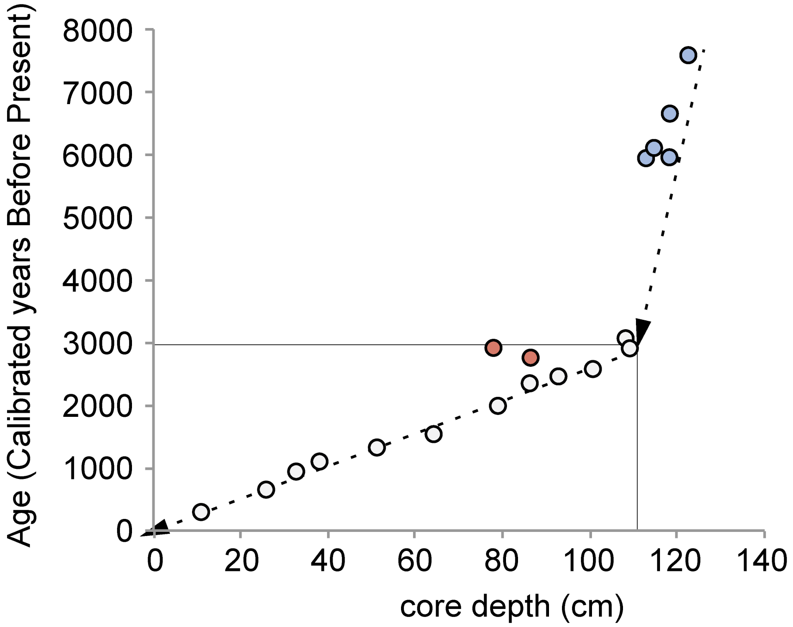

Figure S2. Age-depth plot of radiocarbon results from BLWD-C2. The dates plotted are the highest probability 1σ calibrated result from Calib 7.0. Grey data points plotted were used to generate a final age model in *Bacon* (v2.2) using Bayesian statistical methods (see Fig. 2 in article). The red data points are likely terrestrial plant macrofossils that resided on the terrestrial surface for several hundred years before becoming deposited into Blackwood Sinkhole, and younger results were obtained from other co-stratigraphic terrestrial plant macrofossils (e.g., 86 to 87 cm). Blue data points indicate that sedimentation in Blackwood Sinkhole was negligible or episodic (hiatus between 3.0 and 6.0 ka?) prior to 3000 Cal yrs BP, after which sedimentation was linear and continuous through the late Holocene (least squares regression: r^2=0.991, n = 11 AMS dates). Note: the size of datapoint plotted above mostly encompasses the uncertainty for highest probability 1σ calibration result, see Table S1 for complete radiocarbon calibration results.

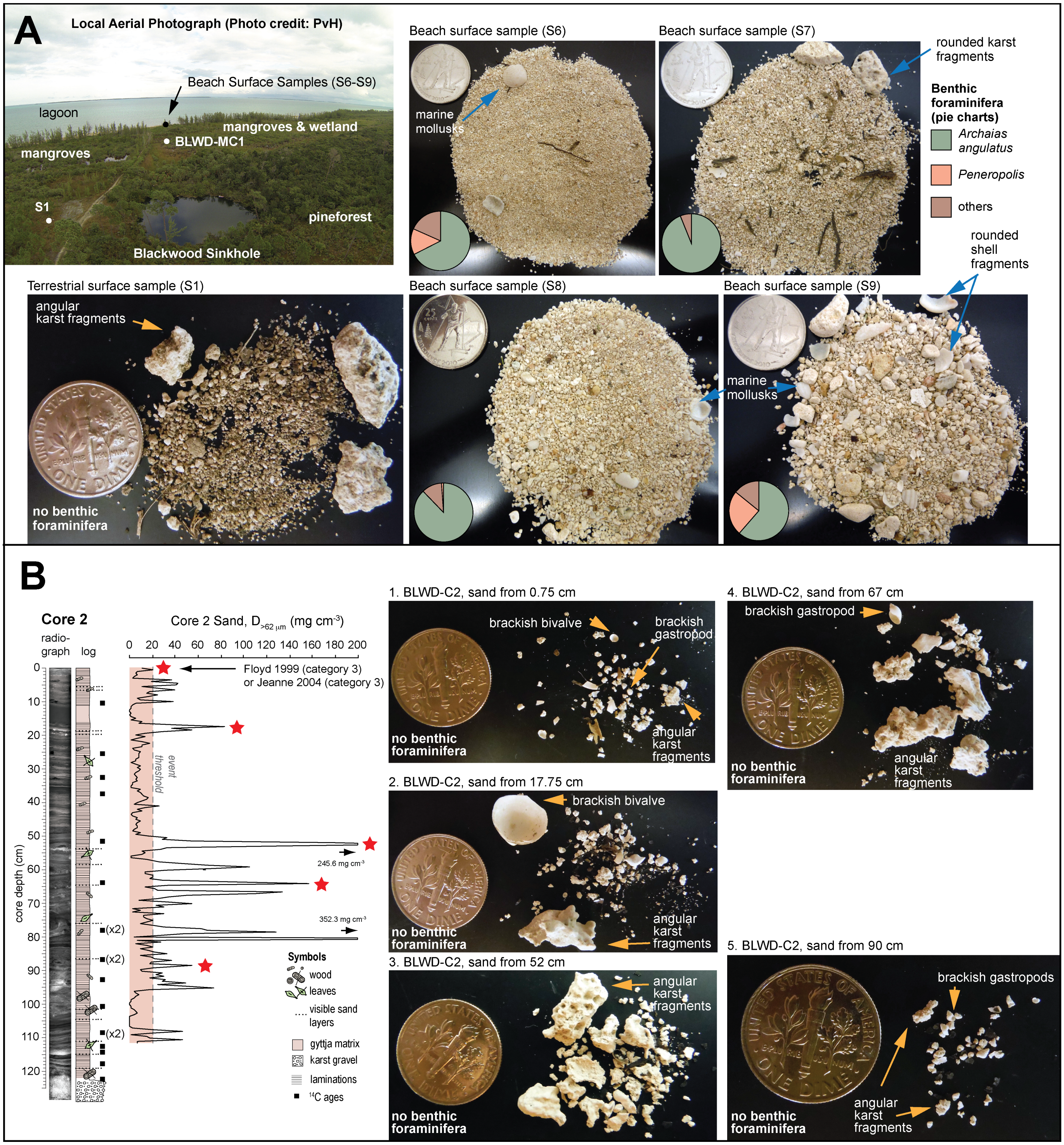

Fig S3. A: An aerial photograph in the top-left depicts the location of surface sediment samples, and a core collected from the deepest location in the adjacent wetland (BLWD-MC1, Table S1). **B:** Detailed analysis of the coarse sediment particles preserved in five coarse-grained layers in BLWD-C2 (red stars, sieved at >63 µm, 1.25 cm^3 sediment volume). The coarse-grained layers are characterized by highly angular and weathered carbonate fragments, whole and articulated brackish mollusks (bivalves, gastropods), and no benthic foraminifera. In the multiple samples examined under stereomicroscopy for microfossils from BLWD-C2, only rare mangrove benthic foraminiferal taxa (e.g., *Rosalina globularis*,

Jadammina macrescens) or brackish testate amoebae were observed (e.g., *Centropyxis aculeata*), which are not in situ because these taxa cannot survive in the anoxic state of the bottom water (saline groundwater). These results indicate that the adjacent terrestrial and wetland environments are the primary source of coarse-grained sediment deposited into Blackwood Sinkhole during intense flooding events. The diameter of an American dime is 17.9 mm, and the diameter of a Canadian quarter is 23.8 mm.

Table S1. Radiocarbon results for core BLWD-C1 and BLWD-C2 (enclosed on page 6 of online supplement). Note that the radiocarbon results from the leaf fragment from BLWD-C2 77.5 to 78 cm, and the twig from BLWD-C2 86.0 to 86.5 cm were excluded from the final Bacon Age model as they were systematically older than the radiocarbon results on material at the same or nearby stratigraphic levels.

Index No.	Lab number	Core	Core interval (cm)	Material	Conventional ^{14}C age	Fraction Modern ($F^{14}C$)	$\delta^{13}C_{org}$ (‰)	Calibrated 1σ ranges (probability)	Calibrated 2σ ranges (probabability)
1	OS-92769	BLWD-C2	10.5 to 11 cm	single leaf	205 ± 25	0.975 ± 0.0032	–27.7	0 to 10 (0.1714)<br>150 to 173 (0.4501)<br>178 to 184 (0.0586)<br>273 to 294 (0.3197)	0 to 15 (0.1552)<br>145 to 214 (0.5482)<br>267 to 301 (0.2965)
2	OS-92835	BLWD-C2	25 to 26 cm	leaf fragments	680 ± 25	0.9188 ± 0.0031	–27.76	570 to 580 (0.2671)<br>**651 to 670 (0.7328)**	563 to 59 (0.3392)<br>**639 to 677 (0.6607)**
3	OS-92771	BLWD-C2	32.5 to 33 cm	leaves	1000 ± 30	0.8828 ± 0.0032	–27.7	834 to 841 (0.0707)<br>**909 to 958 (0.9293)**	798 to 815 (0.0601)<br>822 to 869 (0.1997)<br>898 to 967 (0.7401)
4	OS-90975	BLWD-C2	37.5 to 38 cm	twig	1160 ± 25		–27.24	1007 to 1029 (0.2186)<br>**1053 to 1092 (0.4764)**<br>1106 to 1136 (0.2632)<br>1162 to 1167 (0.0417)	983 to 1034 (0.2638)<br>**1048 to 1151 (0.6654)**<br>1156 to 1171 (0.0706)
5	OS-90976	BLWD-C2	51.25 to 51.75 cm	twig	1380 ± 25		–27.58	**1286 to 1307 (1.)**	**1277 to 1336 (1.)**
6	OS-90995	BLWD-C2	64 to 64.5 cm	twig	1630 ± 30	0.8166 ± 0.0032	–27.21	1420 to 1434 (0.1037)<br>1440 to 1461 (0.1864)<br>**1513 to 1563 (0.7097)**	**1415 to 1572 (0.9473)**<br>1581 to 1602 (0.0526)
7	OS-89451	BLWD-C2	77.5 to 78 cm	leaf	2780 ± 35		–28.88	2810 to 2813 (0.0273)<br>**2845 to 2929 (0.9102)**<br>2936 to 2945 (0.0624)	**2787 to 2957 (1.)**
8	OS-90977	BLWD-C2	78.75 to 79.25 cm	twig	2030 ± 25		–29.1	1934 to 1937 (0.0254)<br>**1945 to 2003 (0.9413)**<br>2029 to 2033 (0.0332)	1899 to 1912 (0.0225)<br>**1921 to 2059 (0.9752)**<br>2099 to 2101 (0.0021)
9	OS-90978	BLWD-C2	86 to 86.5 cm	twig	2610 ± 35		–27.55	**2729 to 2760 (1.)**	2551 to 2555 (0.0034)<br>2618 to 2633 (0.03082)<br>**2705 to 2787 (0.9657)**
10	OS-92771	BLWD-C2	86 to 87 cm	twigs	2290 ± 25	0.752 ± 0.0023		**2313 to 2347 (1.)**	2183 to 2234 (0.2192)<br>**2306 to 2350 (0.7807)**
11	OS-92772	BLWD-C2	92 to 93 cm	twig fragments	2400 ± 30	0.7412 ± 0.0026		2353 to 2376 (0.2327)<br>**2384 to 2459 (0.7672)**	**2346 to 2493 (0.9030)**<br>2600 to 2609 (0.0116)<br>2640 to 2680 (0.0852)
12	OS-90994	BLWD-C2	100 to 101 cm	twig	2520 ± 35	0.7303 ± 0.0032	–15.79	2503 to 2530 (0.1661)<br>**2537 to 2594 (0.4102)**<br>2614 to 2637 (0.1844)<br>2696 to 2727 (0.2391)	**2487 to 2742 (1.)**
13	OS-90996	BLWD-C2	108 to 108.5 cm	twig	2890 ± 35	0.6974 ± 0.0027	–17.29	**2962 to 3072 (1.)**	2891 to 2903 (0.01395)<br>**2924 to 3084 (0.8392)**<br>3086 to 3159 (0.1467)
14	OS-89450	BLWD-C2	108.75 to 109.25 cm	twig	2790 ± 30		–17.59	**2853 to 2928 (0.9729)**<br>2939 to 2941 (0.0270)	2794 to 2831 (0.0910)<br>**2838 to 2960 (0.9089)**
15	OS-92773	BLWD-C2	112.5 to 113 cm	bulk sediment	5160 ± 35	0.5261 ± 0.0024	–31.7	**5900 to 5942 (0.8521)**<br>5972 to 5985 (0.1478)	5761 to 5809 (0.1027)<br>5886 to 5992 (0.8972)
16	OS-92832	BLWD-C2	114.5 to 115 cm	bulk sediment	5280 ± 35	0.5181 ± 0.0021	–32.33	5991 to 6028 (0.2890)<br>6044 to 6068 (0.1698)<br>**6076 to 6118 (0.3330)**<br>6150 to 6177 (0.2079)	5943 to 5971 (0.0879)<br>5985 to 6133 (0.7089)<br>6136 to 6182 (0.2030)
17	OS-92833	BLWD-C2	117.8 to 118.3 cm	bulk sediment	5830 ± 35	0.4836 ± 0.0022	–31.41	6566 to 6588 (0.1757)<br>6602 to 6614 (0.0694)<br>**6615 to 6677 (0.6977)**<br>6705 to 6715 (0.0570)	**6538 to 6737 (1.)**
18	OS-90997	BLWD-C2	117.75 to 118.25 cm	twig	5160 ± 35	0.5263 ± 0.0024	–28.48	**5900 to 5942 (0.8521)**<br>5972 to 5985 (0.1478)	5761 to 5809 (0.1027)<br>**5886 to 5992 (0.8972)**
19	OS-92834	BLWD-C2	122.0 to 122.5 cm	wood fragments	6700 ± 35	0.434 ± 0.002	–29.74	7515 to 7537 (0.3258)<br>**7562 to 7594 (0.6533)**<br>7603 to 7605 (0.0207)	7494 to 7622 (0.9899)<br>7643 to 7650 (0.0100)
20	OS-92767	BLWD-C1	7 to 8 cm	leaf	275 ± 25	0.9665 ± 0.0032	–27.8	**293 to 317 (0.5881)**<br>397 to 422 (0.4118)	157 to 165 (0.0203)<br>**285 to 330 (0.5086)**<br>359 to 429 (0.4710)
21	OS-92768	BLWD-C1	14.5 to 15 cm	leaf fragments	140 ± 25	0.9828 ± 0.003	–25.1	0 to -1 (0.0083)<br>12 to 33 (0.1896)<br>73 to 100 (0.1871)<br>103 to 115 (0.0809)<br>136 to 149 (0.1150)<br>**187 to 225 (0.2792)**<br>254 to 270 (0.1395)	0 (0.0059)<br>7 to 40 (0.1680)<br>60 to 119 (0.2560)<br>122 to 152 (0.1256)<br>**171 to 233 (0.2791)**<br>241 to 280 (0.1651)
22	OS-113156	BLWD-MC1	108-109.5 cm	unidentified plant macrofossil	1490 ± 20	0.8305 ± 0.002	–27.0	1330-1409 (1.)	1351-1390 (1.)
